# Effects of chronic ankle instability on ipsilateral knee cartilage degeneration in mouse model

**DOI:** 10.3389/fmedt.2026.1854284

**Published:** 2026-10-26

**Authors:** Zhi Chen, Fanglu Xu, Bingqian Chen, Zhengfei Wang, Xiaohong Qu, Yao Fu, Jia Yu, Hongtao Zhang

**Affiliations:** 1 Department of Orthopedics, The First Affiliated Hospital of Soochow University, Suzhou, China; 2 Department of Orthopedics, Changshu Hospital Affiliated to Soochow University, Changshu No.1 People's Hospital, Changshu, Jiangsu, China; 3 Department of Nephrology, Affiliated Changshu Hospital of Nantong University, Changshu No.2 People's Hospital, Changshu, Jiangsu, China; 4 School of Physical Education and Sports, Soochow University, Suzhou, Jiangsu, China

**Keywords:** cartilage degeneration, chronic ankle instability, joint coupling, knee osteoarthritis, mouse model

## Abstract

**Objective:**

To explore the effects of chronic ankle instability (CAI) on ipsilateral knee cartilage degeneration and the coupling relationship between the two joints through mouse model.

**Methods:**

Thirty C57BL/6J mice were divided into the Sham group, the CAI group, and the destabilization of medial meniscus (DMM) group. They were respectively subjected to sham surgery, anterior talofibular ligament and calcaneofibular ligament transection, and medial meniscus resection. The degeneration of knee cartilage was evaluated through kinematic experiments, imaging examinations and histopathological analysis within 12 weeks post-operation.

**Results:**

The kinematic experiments exhibited significantly increased passing time and number of slips for CAI group compared to DMM group and Sham group during crossing the balance beam; the step length and step width of CAI group were also larger. Imaging examination revealed obvious osteochondral defects and osteosclerosis of the femoral condyles both in CAI group and DMM group at 12 weeks post-operation. However, the DMM group was more severely affected than CAI group, as its bone mineral density and bone volume fraction were both higher than CAI group. Histopathological analysis exhibited thinner cartilage layer, less chondrocytes and more disordered collagen fibers in DMM group than CAI group at 6 and 12 weeks post-operation. The modified Mankin score and OARSI score of DMM group were also significantly higher than CAI group, while both groups were higher than Sham group.

**Conclusions:**

CAI can cause cartilage degeneration of ipsilateral knee joint in mouse model, there is a coupling relationship between ankle joint and knee joint.

## 1 Introduction

Knee osteoarthritis (KOA) is a common joint disease in clinical practice, often occurring in middle-aged and elderly people, and is characterized by knee pain, stiffness, and limited mobility. Relevant studies have shown that the incidence of KOA among people over 65 years old is as high as over 30% (1–3). It seriously impacts the patient's life and work, and eventually joint replacement surgery was performed as an ending, which bringing heavy medical burden to individuals and society. KOA typically begins with the degeneration of joint cartilage and the loss of cartilage matrix, as well as the progressive development of bone sclerosis and the formation of bone spurs. There are many pathogenic factors for KOA, including age, trauma, inflammation, obesity, etc (4, 5). However, the research on its pathogenesis is still insufficient. Previously, some researches indicated that patients with hip fracture malunion will produce KOA symptoms within a few years (6, 7). However, whether the injury of the lower adjacent joint of the knee joint, the ankle joint, can also cause knee cartilage degeneration is still controversial.

Ankle sprain is one of the most common types of ankle injury in daily life, and more than 70% of them are injuries or ruptures of the lateral ankle ligament complex (8, 9). If it is not properly treated, it is easy to develop into chronic ankle instability (CAI) and cause post-traumatic osteoarthritis (PTOA) of ankle (10). The lateral ligament complex of the ankle joint is composed of the anterior talofibular ligament (ATFL), the calcaneofibular ligament (CFL), and the posterior talofibular ligament (PTFL). These ligaments are thinner and more dispersed than the tenacious deltoid ligament on the medial side of the ankle, which has less traction resistance and is therefore susceptible to injury in the case of excessive ankle varus (11). Among them, the ATFL is the most susceptible to injury, followed by the CFL, while injury of PTFL is extremely rare (12). Some researchers believe that ankle injury will change the force line of the lower limbs, resulting in uneven force on the knee joint and causing the symptoms of KOA (13, 14). While some other researchers believe that ankle injury only causes PTOA of ankle and will not affect the knee joint (15). However, there is still a lack of relevant research data to support these two conclusions, especially basic experiments.

In this study, we intend to explore the potential effect of CAI on knee cartilage degeneration by establishing CAI and destabilization of medial meniscus (DMM) mouse models, and through kinematic experiments, imaging examinations, histopathological analysis. In this study, the DMM model with direct medial meniscus resection and knee joint instability is not set up as a parallel verification group for the same hypothesis as the CAI group, but as a widely recognized classic knee osteoarthritis model, to serve as a severity benchmark for cartilage degeneration evaluation. This setting allows us to quantitatively compare the similarity and difference of the ipsilateral knee cartilage degeneration effect induced by non-direct knee injury (CAI) under a unified evaluation system, so as to more accurately characterize the degree of cartilage degeneration caused by chronic ankle instability. This study may help to deepen people's understanding of the knee-ankle relationship and provide new ideas for the clinical prevention and treatment of KOA.

## 2 Materials and methods

### 2.1 Experimental animal

Thirty 6-week-old male C57BL/6J mice were purchased from Suzhou Zhenhu Laboratory Animal Company (License Number: SCXK2020-0002). The experimental animals (6 mice per cage) were placed in an environment with a 12-hour light/dark cycle, a temperature of 22 ± 2 °C, and a humidity of 55 ± 10%. The mice had free access to food and water, and their health was checked daily. The mice were acclimated to the environment for 2 weeks before the surgery. The animal experiment was approved by the Ethics Committee of Changshu No.1 People's Hospital (License number: 2023038).

A complete animal experiment flowchart is supplemented as Supplementary Figure 1, which clearly shows the full process of the experiment: 1. 30 mice were acclimated for 2 weeks before surgery, then randomly divided into 3 groups (Sham, CAI, DMM) with 10 mice per group; 2. All 30 mice underwent corresponding surgical interventions, and postoperative care was carried out for 3 consecutive days with penicillin injection to prevent infection; 3. 7 rounds of kinematic experiments (balance beam test and gait analysis) were performed on all 30 mice at 1/2/4/6/8/10/12 weeks post-operation (Before 6 weeks after surgery, the number of mice in each group was 10. After 6 weeks, each group consisted of 5 mice); 4. At 6 weeks post-operation, 5 mice in each group were randomly selected for Micro-CT scanning, then euthanized for knee joint tissue collection and subsequent histopathological processing; 5. At 12 weeks post-operation, the remaining 5 mice in each group were euthanized after finishing the last kinematic test, for Micro-CT scanning and histopathological analysis.

Before the formal experiment, we performed *a priori* power calculation to determine the minimum required sample size: we set alpha = 0.05, beta = 0.2 (the test power is 80%), and calculated based on the effect size data of pre-experiments, confirming that a minimum of 5 valid samples per group can stably detect statistically significant differences between groups.

### 2.2 Surgical procedure

All mice were evenly and randomly divided into three groups. After successful inhalation anesthesia with 3% isoflurane, surgical procedures were performed in a sterile environment. The first group, named Sham, was made a longitudinal incision in front of the right knee joint, the muscle was separated up to the joint capsule but not opened, and then the skin incision was sutured. For the second group, named CAI group, an oblique incision was made on the lateral side of the right ankle joint, the ankle capsule was cut open, the ATFL and CFL were found and cut off, then the skin incision was sutured. The third group was named as DMM group. The same as Sham group, a longitudinal incision was made in front of the knee joint, and the joint capsule was opened, the medial meniscus ligament (MML) and the medial meniscus were removed, and the joint capsule and skin were sutured finally (Figure 1a). As explicitly stated in the Introduction section, the DMM group in this study was set as the positive control with known cartilage degeneration severity, to provide a unified reference benchmark for comparing the degree of knee cartilage injury induced by CAI. After successful modeling and awakening, all the mice were transferred to the cage and provided with normal food and water. Penicillin (200, 000 U per day) was intramuscularly injected three consecutive days after the surgery to prevent infection.

**Figure 1:**
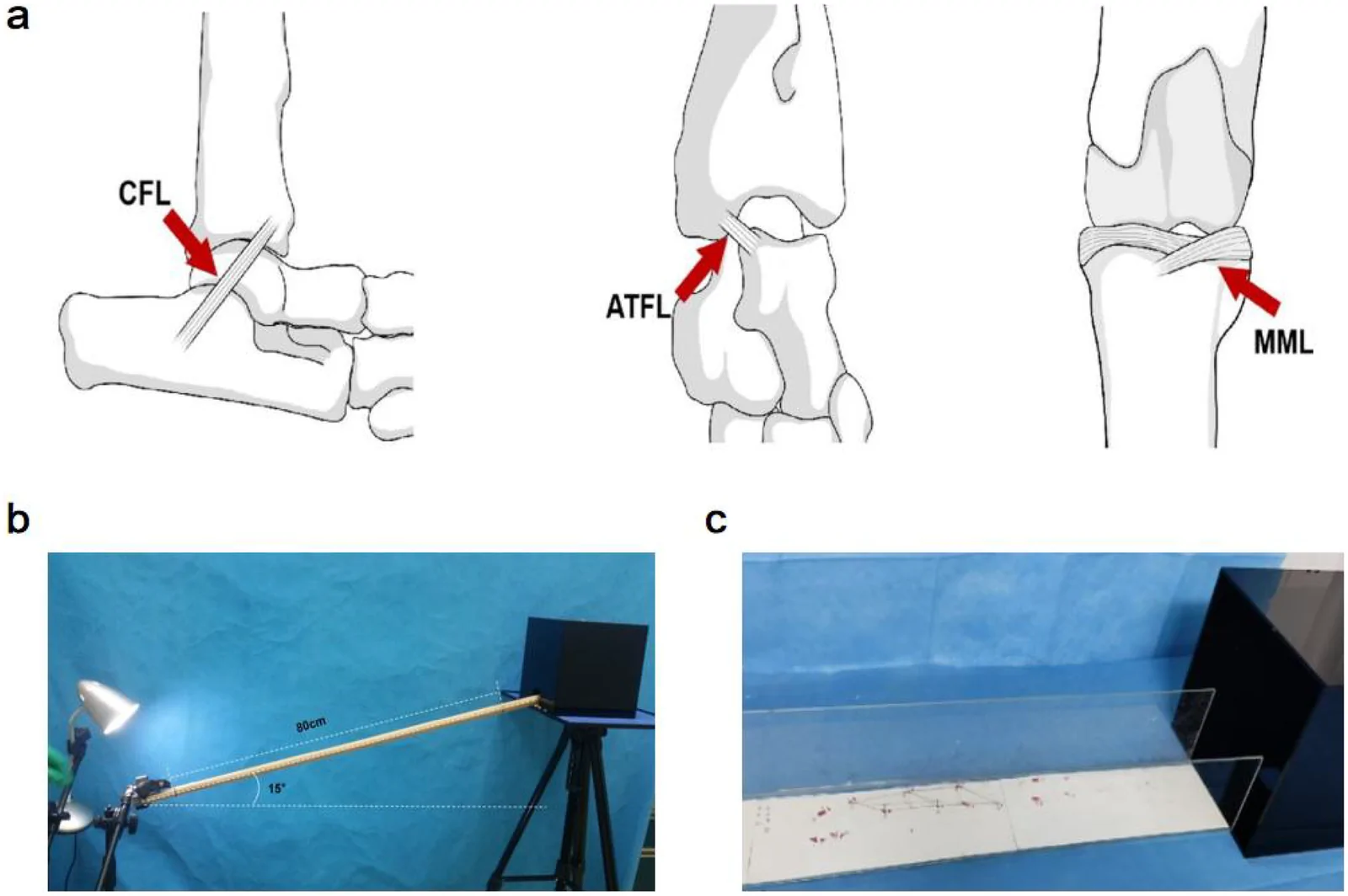
Schematic diagram of the modeling process, balance beam experiment, and gait analysis. **(a)** Schematic diagram of the anatomical position of the calcaneofibular ligament, the anterior talofibular ligament and the medial meniscus ligament; **(b)** Schematic diagram of balance beam experiment; **(c)** Schematic diagram of footprint gait experiment.

### 2.3 Kinematic experiment

The mice were subjected to a balance beam test at week 1/2/4/6/8/10/12 post-surgery to assess their kinematic stability. They were instructed to pass an 80 cm long and 15°slanting wooden pole without interruption, and all slips were recorded, as well as the time to pass through the balance beam (Figure.1b).

A gait analysis experiment was also conducted by recording the footprints left by the mice in a 50 cm × 10 cm × 10 cm (length×width × height) U-shaped transparent channel to observe the significant differences in gait (Figure.1c). The changes were quantitatively described by calculating the step length and step width of the hind foot on the surgical side at 1/2/4/6/8/10/12 weeks after surgery. When analyzing the gait, the first and last footprints on each sheet of paper were removed, and the middle footprints were retained.

### 2.4 Micro-CT scanning

A micro-CT (SkyScan, Belgium) was applied for scanning and evaluating the right knee joint of mice at 6 and 12 weeks post-operation. The parameters were set as voltage 50 kV, current 200 mA, filter plate 0.5 mm Al, and resolution 9 μm. After the scanning, 30 consecutive regions of interest (ROI) were selected from the reconstructed images and quantitative analysis was conducted on the selected knee joint area to calculate the bone mineral density (BMD) and bone volume fraction (BVF). Five mice per group were examined at each time point (6 and 12 weeks post-operation). The ROI was located at the distal femoral condyle, and BMD (mg/cm^3^) and BVF (%) were calculated from the reconstructed images using the software provided with the micro-CT system.

### 2.5 Histopathological analysis

After euthanizing with 200 mg/kg pentobarbital sodium intra-peritonelly, the right knee joints of mice were harvested, followed by fixing in 4% paraformaldehyde for 48 h at 6 and 12 weeks post-operation. The specimens were decalcified with 10% EDTA solution (pH = 7.4) for 1 month, and dehydrated by gradient concentrations of alcohol. Next, the specimens were immersed in n-butanol for 10 h and then embedded in paraffin for 7 h. A semi-automatic paraffin sectioning machine (Leica, Germany) was employed to cut the paraffin blocks into 6-*μ*m thick sagittal sections. Then, the slices were stained with Hematoxylin-Eosin (H&E), Safranin O-Fast Green and immunohistochemical staining of type II collagen to evaluate the changes of the knee joint, and the the progression of KOA was further assessed using the modified Mankin scoring system and OARSI scoring system (16, 17). Quantitative histomorphometric analysis was performed on the stained sections of the medial femoral condyle using ImageJ software. The following parameters were measured: (i) chondrocyte density, defined as the number of chondrocytes per unit area of cartilage; (ii) cartilage thickness and total cartilage area; (iii) the number and depth of articular surface depressions; and (iv) the Safranin O-positive area ratio, defined as the area of positive Safranin O staining relative to the total cartilage area, which was used to quantify proteoglycan content and the “blank areas”. Measurements were performed on five sections per animal by two independent observers blinded to the group allocation. In addition, the following operational definitions were applied: “cartilage wear” was defined as focal loss of the superficial cartilage layer accompanied by surface irregularity and reduced Safranin O staining; chondrocyte apoptosis was defined as chondrocytes with condensed or pyknotic nuclei and lacunar emptying; necrosis was defined as cell loss with cytoplasmic disintegration; and inflammatory cell infiltration was defined as the presence of inflammatory cells within the cartilage, synovium, or subchondral region.

### 2.6 Statistical analysis

Statistical analysis was performed using SPSS 27.0 and GraphPad Prism 10.0. The normality and homogeneity of variance of the data were tested using the Shapiro–Wilk test and Levene's test, respectively. For continuous data (step length, step width, BMD, and BVF), one-way analysis of variance (ANOVA) was applied, followed by Tukey's HSD *post-hoc* test for multiple pairwise comparisons among the three groups. For discrete or ordinal data (number of slips, OARSI score, and modified Mankin score), the Kruskal–Wallis H test followed by Dunn's *post-hoc* test was used. For the longitudinal balance beam data (passing time), a two-way repeated-measures ANOVA with group and time as factors was performed, followed by Bonferroni-corrected pairwise comparisons. Individual data points are shown in all figures together with the mean ± SD. All data were expressed as mean ± standard deviation (Mean ± S.D.). *P* < 0.05 was considered as statistically significant.

## 3 Results

### 3.1 Balance beam experiment

In the balance beam test, there was no significant difference in the passing time and number of slips among the three groups before surgery. From the first week after surgery, the passing time and the number of slips were significantly increased in the CAI and DMM groups than in the Sham group (Figures 2a,b). With the extension of time, the passing time and the number of slips in the CAI group were significantly greater than those in the DMM group. After 8 weeks, a clear plateau phase could be observed for both the CAI group and the DMM group. At 12 weeks after surgery, the passing time in the CAI group was 2.84 times longer than that in the Sham group (P＜0.01), and the number of slips was 19.15 times higher than that in the Sham group (P＜0.01). Meanwhile, the passing time of the DMM group was 1.78 times longer than that of the Sham group (P＜0.05), and the number of slips was 7.27 times higher than that of the Sham group (P＜0.01).

**Figure 2:**
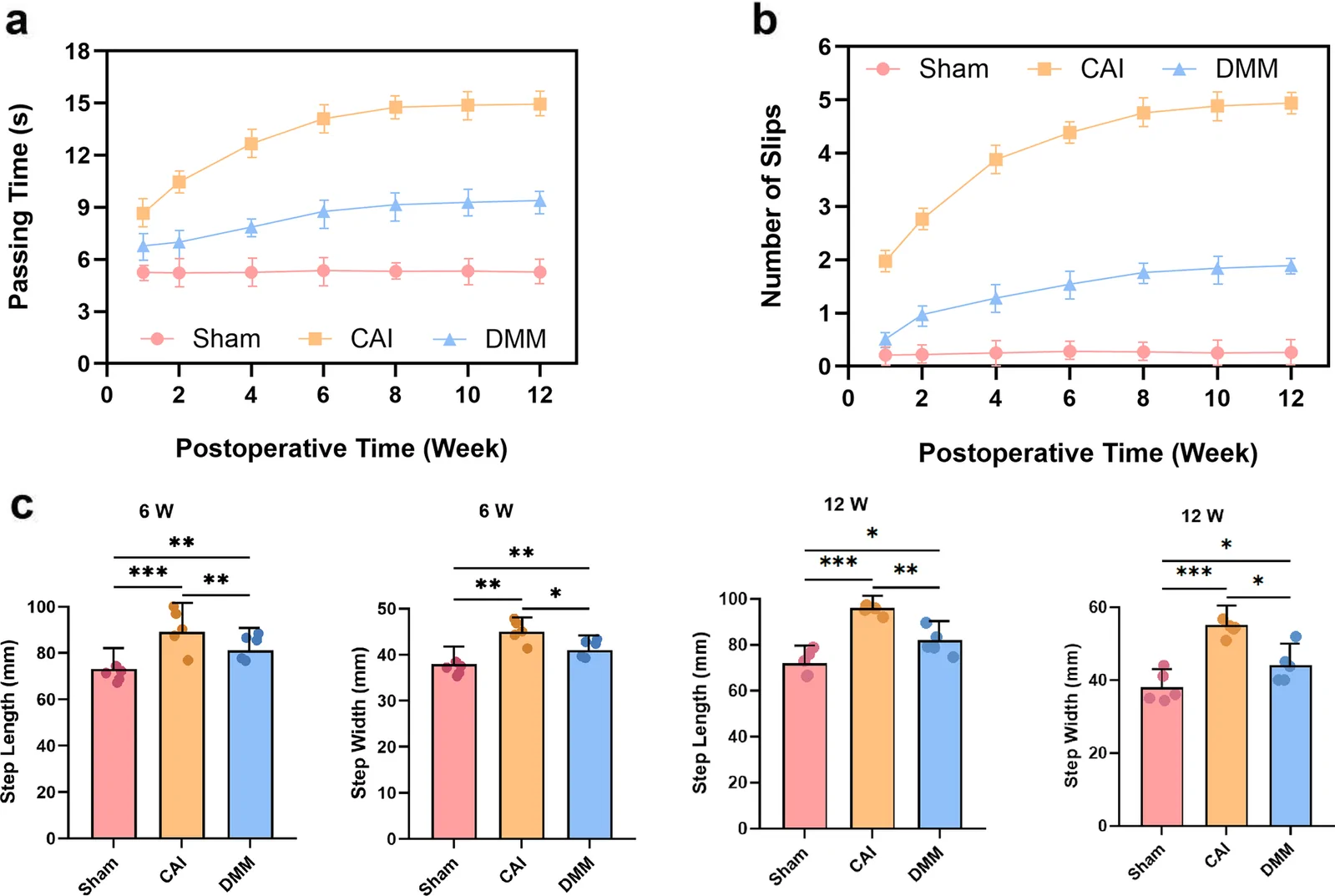
Kinematic experiment results of different groups. **(a)** Time of passing through the balance beam; **(b)** Number of slips while passing the balance beam; **(c)** Step length and step width at 6 weeks and 12 weeks after the operation (*n* = 5, *p < 0.05, **p < 0.01, ***p < 0.001). Individual data points are shown together with the mean ± SD.

Two-way repeated-measures ANOVA revealed significant main effects of group and time, as well as a significant group   ×  time interaction, on passing time (group: F(2, 12) = 22.47, *P* < 0.001; time: F(6, 72) = 13.65, *P* < 0.001; group   ×  time: F(12, 72) = 4.21, *P* < 0.001).

### 3.2 Footprint analysis

The results of gait analysis showed that the step length and step width were significantly increased in the CAI and DMM groups than in the Sham group at 6 weeks after surgery, and more obvious in the CAI group than in the DMM group (Figure 2c). By 12 weeks after surgery, the difference was further amplified. Compared with Sham group, CAI group increased step length by 34.31% (P＜0.001), step width by 45.32% (P＜0.001), DMM group increased step length by 14.09% (P＜0.05), step width by 15.93% (P＜0.05).

### 3.3 Micro-CT examination

Typical images showed that some fine cracks appeared on the surface of the femoral condyle in the CAI group at 6 weeks post-operation, but the structure remained intact. By 12 weeks post-operation, these cracks further expanded, forming bone defects. At the same time, bone hardening and marginal osteophyte formation could be observed. The DMM group was similar to the CAI group but more severe. At 6 weeks post-operation, obvious bone defects could be found in the femoral condyle of the DMM group, and the edge of the bone cortex was hardened. By 12 weeks post-operation, the cartilage and bone in the DMM group were severely worn, with obvious hardening bands formed on the bone surface and osteophytes developed. These phenomena indicated obvious osteoarthritis manifestations (Figure 3a).

**Figure 3:**
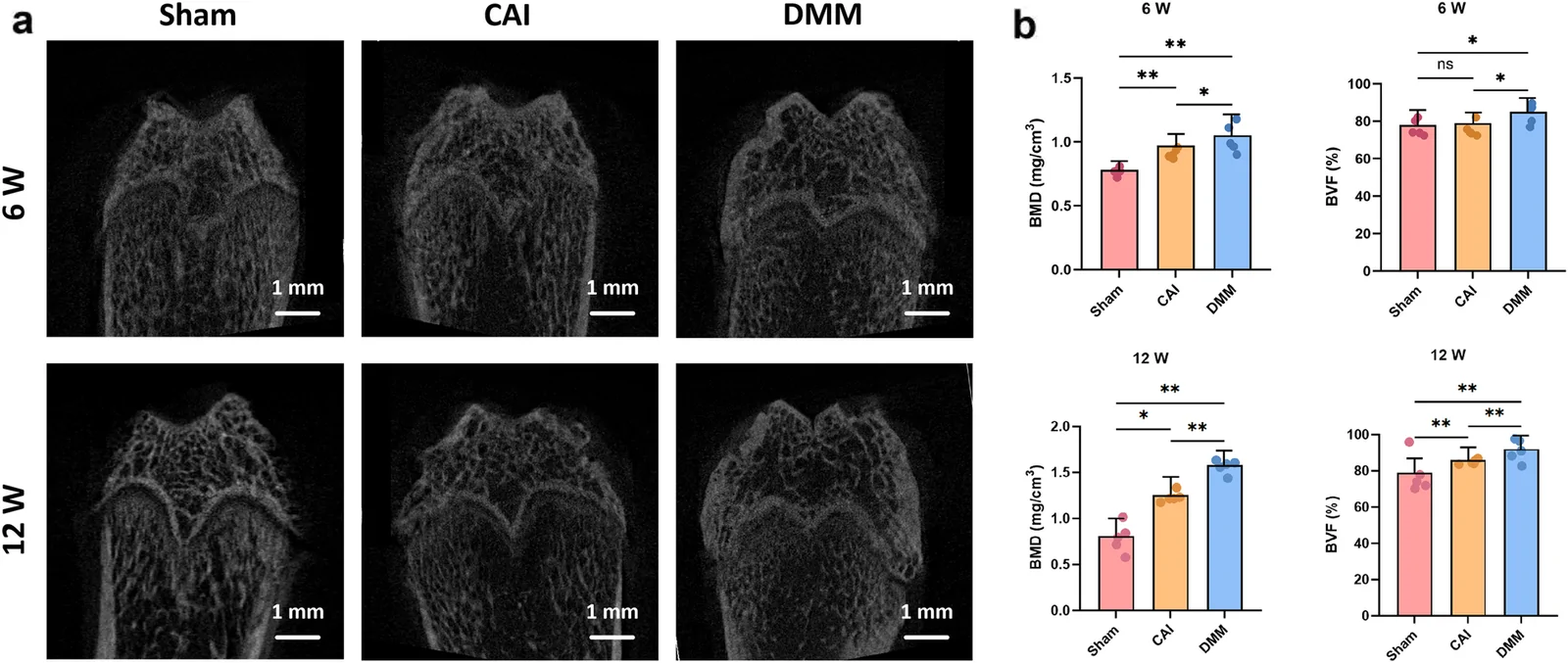
Imaging examination of knee joints for different groups. **(a)** Micro-CT images of knee joints in different groups at 6 and 12 weeks postoperative (scale bar: 1 mm); **(b)** Calculation of BMD and BVF for knee joints based on micro-CT scanning data (*n* = 5, *p < 0.05, **p < 0.01, ns *p* > 0.05). Individual data points are shown together with the mean ± SD.

As the postoperative time extended, the BMD and BVF of the CAI group and the DMM group both significantly increased and were higher than those of the Sham group. At 12 weeks after the surgery, the BMD of the CAI group was 1.54 times that of the Sham group (P＜0.05), while DMM group was 1.95 times than Sham group (P＜0.01). The BVF of the CAI group was 1.19 times than the Sham group (P＜0.01), while that of the DMM group was 1.26 times than the Sham group (P＜0.01). The quantitative results showed that both the CAI group and the DMM group exhibited osteosclerosis of the knee joint, and the DMM group was more severe (Figure 3b).

### 3.4 Histological assessment

The H&E staining results showed that the joint surfaces of the Sham group were smooth and intact, without obvious defects or degeneration. At 6 weeks after the operation, there was some wear on the articular surface in the CAI group, but the arrangement of chondrocytes was still relatively neat. For the DMM group, the joint surface lost its integrity, cartilage wear occurred, and the number of chondrocytes decreased. By 12 weeks after the operation, the articular surface in the CAI group also lost its flatness, the staining of the cartilage matrix became lighter, the arrangement of chondrocytes was disordered, and some chondrocytes apoptosis were captured. For the DMM group, large areas of cartilage and bone defects on the joint surface could be observed, with a significant reduction in the number of chondrocytes, obvious cell necrosis, and infiltration of inflammatory cells (Figure 4a). The term “cartilage wear” is operationally defined in the Methods.

**Figure 4:**
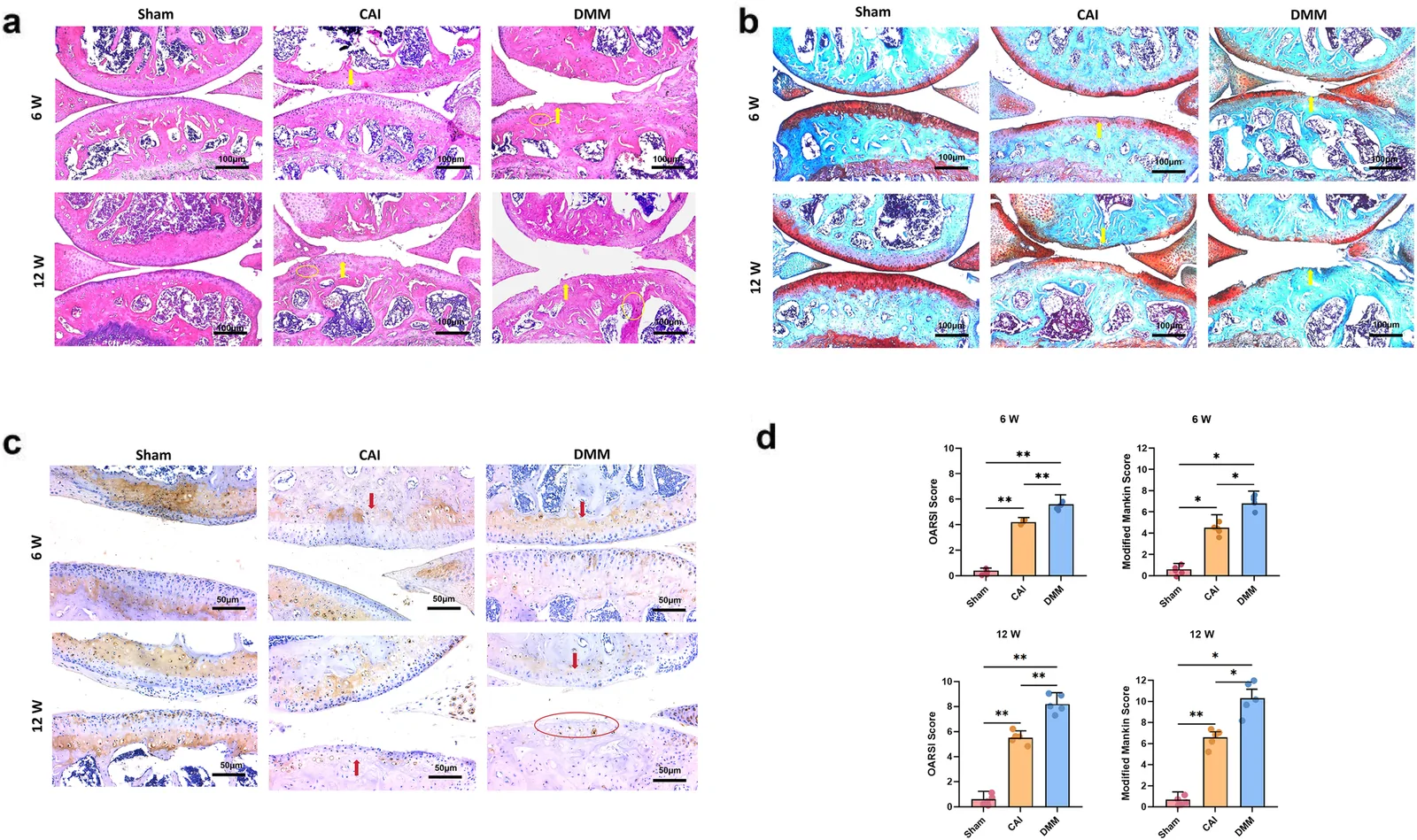
Histological analysis of knee joints for different groups. **(a)** H&E staining results of knee joint samples at 6 and 12 weeks postoperative, with arrows indicate the key pathological features, including articular surface irregularity and depression, chondrocyte loss, and matrix loss; circles indicate cell necrosis and apoptosis (scale bar: 100 μm). **(b)** Safranin O-Fast Green staining results of knee joint samples at 6 and 12 weeks postoperative, with arrows indicating reduced Safranin O staining (blank areas) and matrix damage (scale bar: 100 μm). **(c)** Immunohistochemical staining of type II collagen results of knee joint samples at 6 and 12 weeks postoperative, with arrows indicate the thinning, lightening and fibrosis of the cartilage matrix layer; circle represents the defect of joint surface (scale bar: 50 μm). **(d)** OARSI score and modified Mankin score for different groups at 6 and 12 weeks postoperative (*n* = 5, *p < 0.05, **p < 0.01). Individual data points are shown together with the mean ± SD.

The results of Safranin O-Fast Green staining showed that the chondrocytes in the Sham group were uniformly stained, with distinct cartilaginous and bony layers, presenting a typical manifestation of surface transition layer—radiation layer—calcification layer. In the CAI group, at 6 weeks after the operation, the staining of the cartilage layer became lighter and local depressions on the surface could already be observed. In the DMM group, in addition to the lightening of the staining, the thickness of the cartilage layer also significantly decreased, and the local matrix was damaged. At 12 weeks after the operation, the Safranin O staining in the CAI group became lighter and uneven, with local blank areas appearing. The hierarchical structure of the cartilage was disordered, and the green collagen fibers were arranged disorderly. In the DMM group, large areas of blank areas stained with safranin-O appeared, collagen fibers showed fissures and shedding, the number of chondrocytes decreased significantly, and the destruction of cartilage matrix was obvious (Figure 4b). The lighter Safranin O staining observed in the CAI and DMM groups reflects a biological phenotype of proteoglycan depletion rather than a technical staining artifact: all sections were stained in the same batch under identical conditions, the lighter staining was consistently localized to the superficial and middle zones of the cartilage, and it progressively worsened from 6 to 12 weeks post-operation.

Furthermore, the high-magnification immunohistochemical staining images of type II collagen also showed similar results. As the postoperative period extended, the staining areas and thickness of type II collagen in the CAI group and DMM both significantly decreased, indicating the loss of cartilage matrix. At the same time point, the performance of the DMM group was more severe than that of the CAI group. At 12 weeks after the surgery, the staining of type II collagen in the DMM group was extremely light and could hardly be detected (Figure 4c).

At 6 weeks and 12 weeks after the surgery, whether the modified Mankin score or OARSI score of the CAI group and the DMM group were both significantly higher than those of the Sham group. As time went on, the score of both groups gradually increased, indicating a tendency towards osteoarthritis. It is worth noting that the modified Mankin score and OARSI score of the DMM group were both higher than those of the CAI group at two time points post-operation, which was consistent with the staining results (Figure 4d).

Quantitative histomorphometric analysis of the medial femoral condyle corroborated these qualitative observations (Figure 5). Compared with the Sham group, chondrocyte density was significantly reduced in both the CAI group and the DMM group at 6 and 12 weeks post-operation, with the most pronounced reduction in the DMM group. Similarly, cartilage thickness and total cartilage area were decreased in both groups at the two time points, indicating progressive thinning and loss of the articular cartilage. Consistently, the number and depth of articular surface depressions were increased in the CAI and DMM groups, which, together with the reduced cartilage thickness, objectively reflected the deterioration of articular surface flatness. Furthermore, the Safranin O-positive area ratio was significantly reduced in both groups, confirming the proteoglycan depletion and matrix damage (blank areas) observed on Safranin O-Fast Green staining. For all parameters, the changes were most severe in the DMM group at both time points, consistent with the qualitative staining findings and the modified Mankin and OARSI scores.

**Figure 5:**
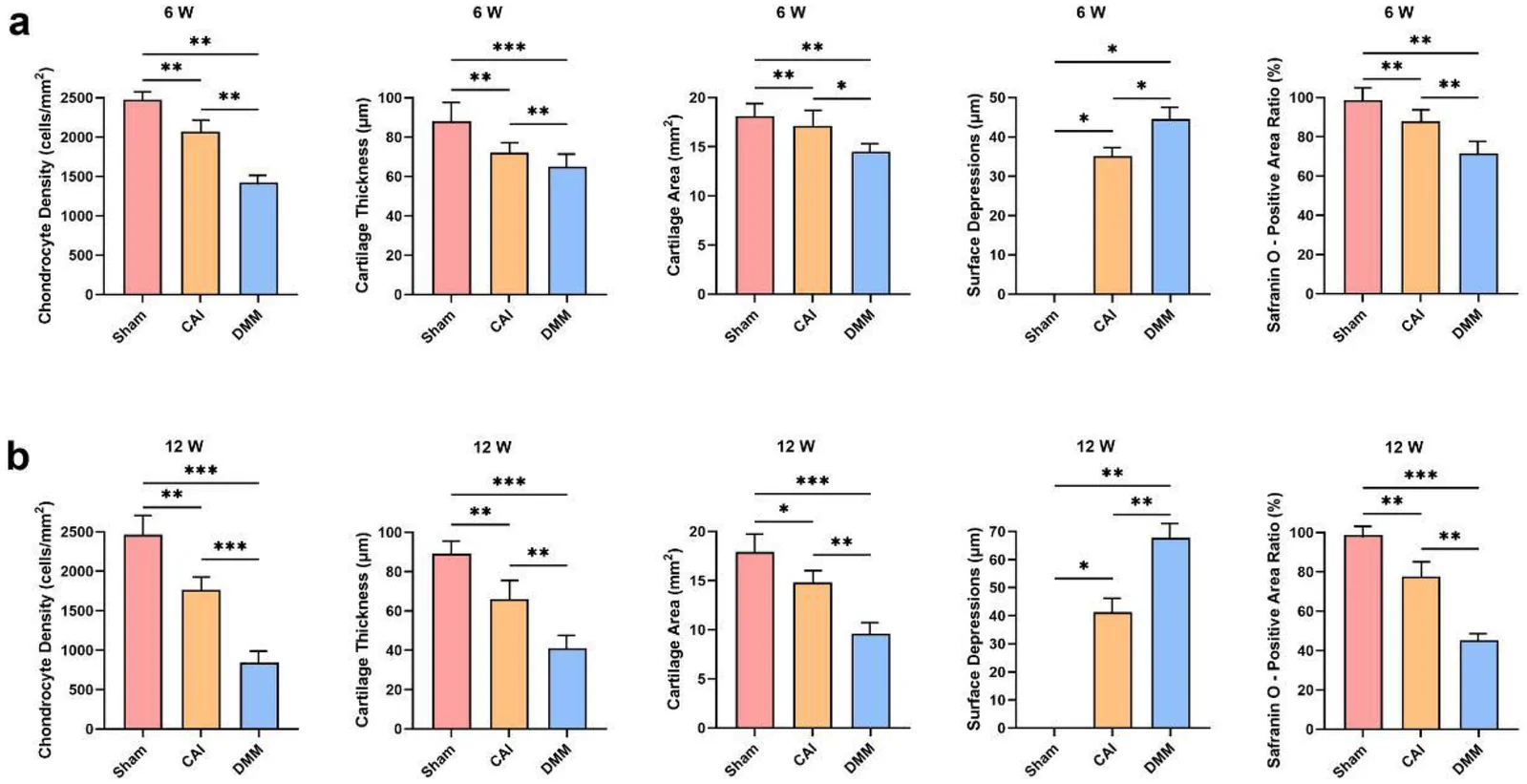
Quantitative histomorphometric analysis of chondrocyte density, cartilage thickness, cartilage area, number and depth of surface depressions, and safranin O-positive area ratio (*n* = 5, *p < 0.05, **p < 0.01, ***p < 0.001).

## 4 Discussion

Limitations still remain in current understanding of the coupling relationship between the knee joint and the ankle joint in clinical practice. Factually, as two adjacent joints that maintain lower limb movement functions, the ankle joint and the knee joint have a close connection in terms of kinematics (18, 19). In order to verify the coupling relationship between the knee joint and the ankle joint, this present study explored the impact of CAI on the degeneration of ipsilateral knee cartilage through a series of experimental methods including kinematic tests, imaging examinations, and histopathological analysis by establishing mouse models of ankle instability and knee instability.

Regardless of the passing time or the number of slips, both the CAI group and the DMM group showed a gradually increasing trend, suggesting that the motor and balance abilities of the mice in both groups were to some extent impaired. The appearance of the plateau in the curve graph indicates that possibly after 8 weeks, the proliferation of scar tissue around the joints compensated for some of the joint stability. From the balance beam experiment and gait analysis, it can be inferred that the stability of the ankle joint may have a greater impact on maintaining normal walking function and gait than the knee joint, which was consistent with the research results of *Taylor et al* (20). For the CAI group, since the lateral ligament complex of the ankle joint was injured, the foot usually presents an outward swinging gait, so the step length and step width significantly increase (21). For the DMM group, according to previous reports, since the medial meniscus was removed, the knee joint bends inward, causing the lower limb force line to move axially inward, and the ankle joint also performs an eversion compensation (22). Therefore, the step length and step width also increase, but are smaller than those of the CAI group. The results of the above kinematic experiments indicate that there is a coupling relationship between the ankle joint and the knee joint. To maintain normal walking function and gait, the adjacent two joints influence and compensate for each other.

The most significant pathophysiological changes in KOA are the damage and degeneration of articular cartilage, along with the accompanying osteosclerosis and osteophyte formation, especially on the femoral side (23). This study conducted a direct assessment of the state of bone and cartilage in the mouse knee joint through imaging and histopathological methods. The results of micro-CT showed that both the CAI group and the DMM group had varying degrees of cartilage injury and bone defect, whether at 6 weeks or 12 weeks after the operation. Among them, the degree of injury and degeneration in the DMM group was the most severe. The BMD and BVF calculated based on micro-CT were consistent with the results of imaging. Compared with the Sham group, the BMD and BVF in the DMM group and the CAI group were significantly increased, and those in the DMM group were higher than those in the CAI group. This suggests that the bone of the knee joint in the DMM group and the CAI group has hardened and there are manifestations of osteoarthritis. The above results suggest that ankle instability can indirectly affect the knee joint. We speculate that after the lateral stabilizing structure of the ankle joint is damaged, the talus may shift inward, which may potentially cause a slight inward deviation of the lower limb force line from bottom to top, and further lead to the possible uneven distribution of load in the medial compartment of the knee joint, thus inducing progressive cartilage wear and subsequent degeneration during movement. At present, this inference is only a reasonable hypothesis based on the existing kinematic and imaging results, and we have not carried out direct biomechanical loading test in this study to confirm this potential mechanism. We will further conduct biomechanical experiments to verify this point. Of course, this kind of impact is less severe than the degree of degeneration caused by knee instability resulting from direct removal of the medial meniscus. *Selcuk* et al. 's research indicates that even under normal physiological conditions, the pressure on the medial and lateral compartments of the knee joint is unbalanced (24). The pressure in the medial compartment is usually greater than that in the lateral compartment. Therefore, the degeneration of knee cartilage often starts from the medial side and gradually spreads to the condylar fossa and the lateral compartment (25, 26). This is also the reason why genu varum is much more common than genu valgum. Our research results also confirm this point.

The results of histopathology showed that both the CAI group and the DMM group had varying degrees of chondrocyte apoptosis and extracellular matrix loss, with the DMM group having a more severe situation than the CAI group. All these are manifestations of cartilage tissue degeneration. It is worth noting that the integrity of the tidemark in the DMM group was damaged at 6 weeks after the operation, indicating that the injury involved the subchondral bone layer. In contrast, the damage to the tidemark occurred in the CAI group at 12 weeks after the operation. Subchondral bone is the boundary between cartilage tissue and bone tissue, playing a series of important roles such as supporting articular cartilage, providing nutrients and participating in cell metabolism (27–30). These findings suggest that CAI can cause similar histopathological effects as DMM, but to a lesser extent than the DMM group at the same period. The modified Mankin score and OARSI score are commonly used scoring tools for evaluating the degeneration of articular cartilage (31, 32). It can also be found from the scoring results that cartilage degeneration occurred in both the CAI group and the DMM group, and the score of the CAI group was often lower than that of the DMM group during the same time point. This result indicates that ankle instability can affect the cartilage of the ipsilateral knee joint, but this impact is indirect and slow, and it is not as fast and severe as those caused by joint degeneration resulting from the destruction of the stable structure of the knee joint itself (such as the removal of the medial meniscus).

This study also has certain limitations. Firstly, although numerous studies have revealed that both humans and mice belong to the plantigrade group and their ankle and knee joint anatomical structures are very similar and comparable, caution should be exercised when directly extending the conclusion of this study to the human body (33, 34). Further research is necessary to confirm its applicability, and this study merely provides reference and inspiration for clinical research. Secondly, the sample size of this study is limited, and more in-depth direct biomechanical loading measurement experiments and cytological experiments are needed to further improve the research process. Thirdly, only one sham procedure (knee sham) was used as the control for the two surgical interventions. We acknowledge that an additional sham operation on the ankle (exposure of the ankle joint capsule without transection of the ATFL/CFL) would more rigorously exclude any effect of the surgical exposition itself. Nevertheless, given that the sham group showed no significant changes in any kinematic parameter and that the kinematic impairment in the CAI group progressed gradually over 12 weeks, we consider it unlikely that the observed phenotype resulted from the surgical procedure alone.

## 5 Conclusion

In this study, by establishing CAI and DMM mouse models and through methods including kinematic experiments, imaging examinations, and histological analyses, we initially clarified the possible effects of CAI on the degeneration of knee joint cartilage, and revealed the coupling relationship between the ankle joint and the knee joint in maintaining the movement function of the lower limbs. This study is expected to promote and deepen people's understanding of the knee-ankle relationship, and we hope it can provide new ideas and references for the clinical prevention and treatment of KOA.

## Data Availability

The original contributions presented in the study are included in the article/Supplementary Material, further inquiries can be directed to the corresponding authors.
