# Supplementary material for: Effects of chronic ankle instability on ipsilateral knee cartilage degeneration in mouse model
Source: Front Med Technol. 2026 Oct 26;8:1854284. doi: 10.3389/fmedt.2026.1854284 (PMC13745472; doi:10.3389/fmedt.2026.1854284)
Supplement: Supplementary file 1 [file Image1.pdf]

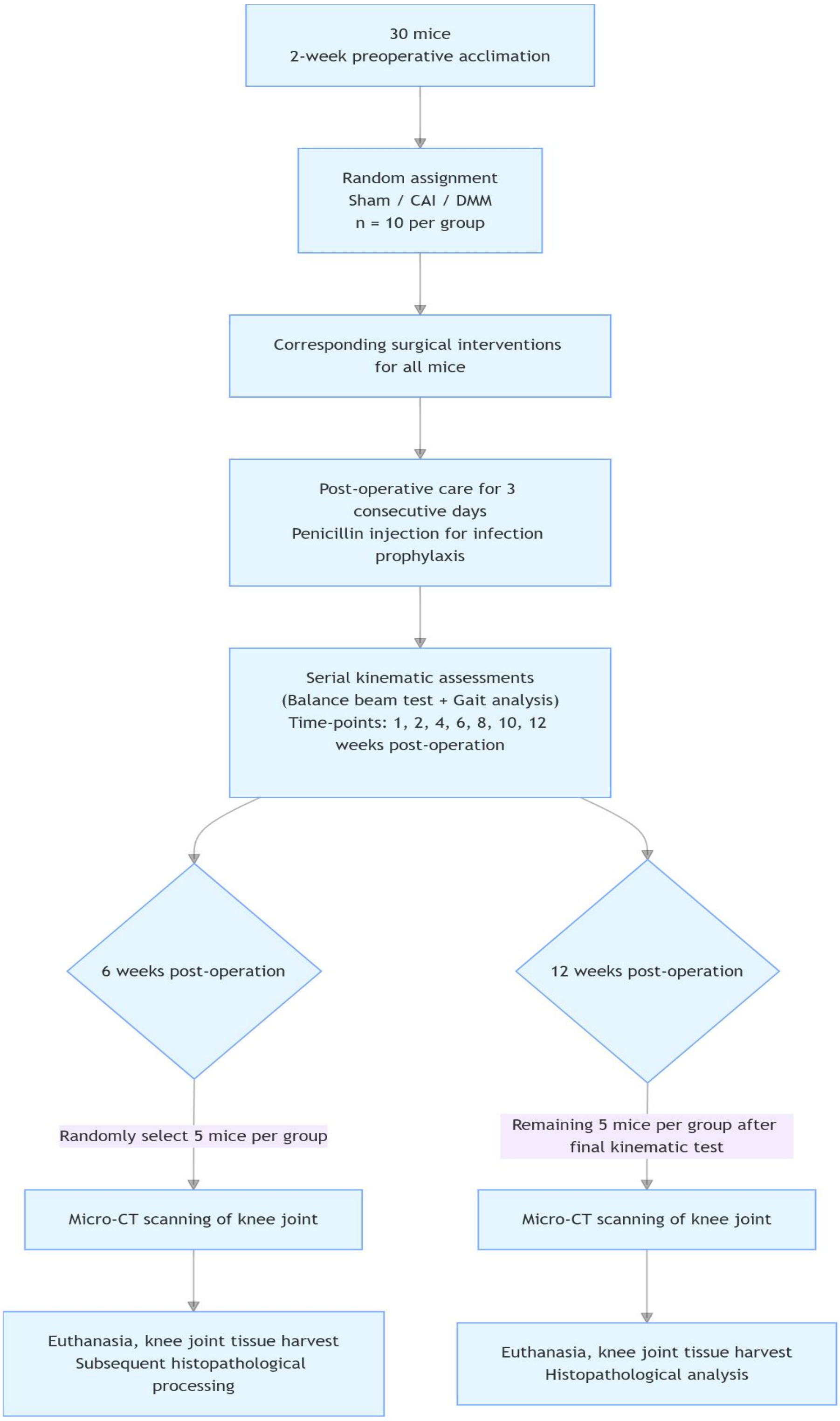


Supplementary Figure 1. Schematic diagram of the whole process of experimental animal assignment.
